# Prognostic factors for non-success in patients with sciatica and disc herniation

**DOI:** 10.1186/1471-2474-13-183

**Published:** 2012-09-22

**Authors:** Anne Julsrud Haugen, Jens Ivar Brox, Lars Grøvle, Anne Keller, Bård Natvig, Dag Soldal, Margreth Grotle

**Affiliations:** 1 Department of Rheumatology, Østfold Hospital Trust, Fredrikstad, Norway; 2 Section for Back Surgery and Physical Medicine and Rehabilitation, Orthopaedic Department, Oslo University Hospital, Rikshospitalet, Oslo, Norway; 3 Department of Physical Medicine and Rehabilitation, Oslo University Hospital, Ullevål, Oslo, Norway; 4 National Resource Centre for Rehabilitation in Rheumatology, Department of Rheumatology, Diakonhjemmet Hospital, Oslo, Norway; 5 Department of General Practice, Institute of Health and Society, University of Oslo, Oslo, Norway; 6 Department of Rheumatology, Sørlandet Hospital Health Enterprise, Kristiansand, Norway; 7 FORMI (Communication Unit for Musculoskeletal Disorders), Division of Neuroscience, Oslo University Hospital, Ullevål, Oslo, Norway

**Keywords:** Sciatica, Disc herniation, Prognostic factors, Non-success

## Abstract

**Background:**

Few studies have investigated prognostic factors for patients with sciatica, especially for patients treated without surgery. The aim of this study was to identify factors associated with non-success after 1 and 2 years of follow-up and to test the prognostic value of surgical treatment for sciatica.

**Methods:**

The study was a prospective multicentre observational study including 466 patients with sciatica and lumbar disc herniation. Potential prognostic factors were sociodemographic characteristics, back pain history, kinesiophobia, emotional distress, pain, comorbidity and clinical examination findings. Study participation did not alter treatment considerations for the patients in the clinics. Patients reported on the questionnaires if surgery of the disc herniation had been performed. Uni- and multivariate logistic regression analyses were used to evaluate factors associated with non-success, defined as Maine–Seattle Back Questionnaire score of ≥5 (0–12) (primary outcome) and Sciatica Bothersomeness Index ≥7 (0–24) (secondary outcome).

**Results:**

Rates of non-success were at 1 and 2 years 44% and 39% for the main outcome and 47% and 42% for the secondary outcome. Approximately 1/3 of the patients were treated surgically. For the main outcome variable, in the final multivariate model non-success at 1 year was significantly associated with being male (OR 1.70 [95% CI; 1.06 − 2.73]), smoker (2.06 [1.31 − 3.25]), more back pain (1.0 [1.01 − 1.02]), more comorbid subjective health complaints (1.09 [1.03 − 1.15]), reduced tendon reflex (1.62 [1.03 − 2.56]), and not treated surgically (2.97 [1.75 − 5.04]). Further, factors significantly associated with non-success at 2 years were duration of back problems >; 1 year (1.92 [1.11 − 3.32]), duration of sciatica >; 3 months (2.30 [1.40 − 3.80]), more comorbid subjective health complaints (1.10 [1.03 − 1.17]) and kinesiophobia (1.04 [1.00 − 1.08]). For the secondary outcome variable, in the final multivariate model, more comorbid subjective health complaints, more back pain, muscular weakness at clinical examination, and not treated surgically, were independent prognostic factors for non-success at both 1 and 2 years.

**Conclusions:**

The results indicate that the prognosis for sciatica referred to secondary care is not that good and only slightly better after surgery and that comorbidity should be assessed in patients with sciatica. This calls for a broader assessment of patients with sciatica than the traditional clinical assessment in which mainly the physical symptoms and signs are investigated.

## Background

Sciatica, also known as nerve root pain, or radiculopathy in the distribution of the lumbosacral nerves, is defined as radiating pain in the leg below knee level [1,2]. A lumbar disc herniation is the most common cause of sciatica [3,4]. Sciatica is associated with more severe pain and disability than is low back pain alone [5]. Most patients receive conservative treatment, and a minority of patients requires surgery [3,6]. The success rates and prognoses for sciatica vary between studies, depending on the inclusion criteria and outcome measures used. A Dutch study on primary care patients indicated a good prognosis for sciatica with approximately 75% of the patients recovering after 3 months [7]. However, in a Finnish study on patients with sciatica who were referred to hospital, nearly 70% had persistent sciatica symptoms 13 years later [8]. The success rates in the Maine Lumbar Spine Study were 80% for surgically treated patients and 56% for non-surgically treated patients at 1 year; and 70% for surgically treated patients and 56% for non-surgically treated patients at 5 years [9,10]. In another large study of sciatica, the SPORT study, approximately 80% of surgically treated patients and 60% of non-surgically treated patients reported major improvement after 1 and 2 years of follow-up [11,12].

Most studies of the prognostic factors for sciatica have been performed on patients who have undergone surgical treatment, and have assessed the influence of sociodemographic, work-related, psychological, imaging, pain-related, surgery-related, and clinical factors [13,14]. In a randomised study on early surgery versus prolonged conservative treatment for sciatica, female sex was found to be a strong predictor of an unsatisfactory outcome in both groups [15].

There is limited knowledge about the prognosis of patients who are treated non-surgically for sciatica. A recent review found no strong or consistent predictor of persistent disability in non-surgically treated patients with sciatica [16]. Symptoms of depression and anxiety were predictors for a worsening of pain and function after up to 3 years of follow-up in the Maine Study [17]. In a cohort of French workers, psychosomatic symptoms, long-lasting duration of sciatica, carrying heavy loads, and driving at least 2 hours per day predicted persistent sciatica after 2 years [18]. The clinical findings of radiculopathy are important for the diagnosis of sciatica [1]. Positive nerve stretch tests have been identified as a predictor of poor outcome in some studies of sciatica [7,15,19]. However, few studies have explored how other clinical signs and symptoms influence the prognosis of sciatica [20].

Our primary aim of the present study was to identify the prognostic factors associated with non-success after 1 and 2 years of follow-up for sciatica and disc herniation in patients referred for secondary care. Our secondary aim was to test the prognostic value of surgical treatment for sciatica.

## Methods

### Design

A prospective observational multicenter cohort study was conducted. The Regional Committee for Medical Research Ethics and The Ombudsmann for Privacy in Research at the Norwegian Social Science Data Services approved the study protocol.

### Cohort selection and recruitment

Patients were recruited from specialty back clinics at four public hospitals in southeast Norway (Sykehuset Østfold, Sørlandet Sykehus, Oslo Universitetssykehus Ullevål and Sykehuset Innlandet). The inclusion period was 2 years, throughout 2005 and 2006. The patients were referred to the specialty back clinics by primary health care services. The participating specialty back clinics were all in public hospitals, and the Norwegian Social Insurance pays for patients referred to public hospitals. The majority (60%) of the patients (n = 280) were recruited from Sykehuset Østfold, which serves a district with 260,000 inhabitants. About 200 patients with sciatica were referred yearly to this specialty back clinic at the start of the study. This suggests that about 3/4 of the patients referred to this hospital for sciatica were included in the study. The next largest population was from Sørlandet Sykehus (n = 89), located in a district with 162,000 inhabitants. This back clinic treated about 120 patients with sciatica yearly, so some more than 1/3 of eligible patients were included in the study. We do not have data on the proportion of eligible patients recruited to the study from the other 2 hospitals, or the number of eligible patients who either were not invited or declined to participate in the study. In the centre that enrolled the majority of the patients, less than 5% of eligible patients declined to participate during the first year of the study.

In each back clinic, consecutive eligible patients were invited to participate in the study by a physician or a physiotherapist.

Inclusion criteria were age ≥18 years, radiating pain and/or paresis below knee level and a disc herniation at the corresponding level and side that had been verified by magnetic resonance imaging (MRI) or computed tomography (CT). Exclusion criteria were prior surgery at the same disc level, fracture, infection, malignancy, pregnancy and lack of fluency in Norwegian. All participants received oral and written information about the study and gave their informed consent to their participation.

Study participation did not involve any specific type of intervention or alter treatment considerations for the patients, nor did it involve any specific advantages or benefits for the patients in the clinics. Hence, patients received the usual consultations including a clinical examination, information about sciatica and disc herniation, back exercises, physical therapy, and pain medication. Information and treatment were given on an individual basis to the patients at each center.

Surgery was performed for patients with severe symptoms. The patient was referred from the back clinic to an orthopedic surgeon, who made the final decision about surgical treatment. The decision about surgery was made for individual patients at each center and no standardized criteria were established for surgical treatment.

### Procedure

At the day of inclusion patients completed a comprehensive questionnaire. Baseline data were collected at the first visit to the department. Clinical examination was conducted by a physician or physiotherapist. A follow-up questionnaire and a prepaid envelope were sent to the patients after 3, 6, 12 and 24 months. A reminder was sent after 2 weeks if no reply was obtained.

### Potential prognostic factors

At inclusion, the following sociodemographic factors were recorded: age, sex, education (years of schooling), smoking status and work status. Patients also reported their history of back pain (dichotomised to <; 1 year or ≥ 1 year), previous episodes of sciatica (0 or ≥ 1), and the duration of the current episode (dichotomised to <; 3 months or ≥ 3 months).

The following patient-reported variables were also recorded.

Pain intensity in the back and in the leg (sciatica) during the previous week was recorded on a horizontal visual analogue scale (VAS) ranging from 0 (no pain) to 100 (worst pain ever).

The comorbid subjective health complaints inventory included 29 common somatic and psychological complaints, such as muscular pain, headaches, stomach pain and discomfort, hot flushes, extra heart beats, sleep problems, tiredness, dizziness, anxiety and depressed thoughts during the previous month [21,22]. Patients graded the intensity of each complaint during the previous month as not at all (0), a little (1), some (2) or severe (3). Each item was dichotomised to absent (0) or present (1, 2, 3). Because most patients with sciatica have low back pain and leg pain during exercise, the 2 items referring to these symptoms were excluded, resulting in a score in the range of 0–27.

The patients also completed the Tampa Scale for Kinesiophobia (TSK) [23,24], a 13-item, four-point questionnaire. Scores range from 13 to 52, where higher scores indicate increased kinesiophobia.

Emotional distress was assessed using the Hopkins Symptom Check List-25 (HSCL-25) [25]. The questionnaire includes 25 items on depression, anxiety and somatisation during the previous week and ranges from 1 (not at all) to 4 (extremely). The score is calculated as the mean of the completed items. An average item score of ≥ 1.75 was found to be a good predictor of current help-seeking behaviour in a Norwegian epidemiological study and is commonly used to define cases with emotional distress [26].

The sciatica-specific clinical assessment included motor function (deemed abnormal if the extension or flexion of the knee or ankle or the extension of the big toe was reduced; if, when standing on one leg, the pelvis tilted to the side opposite the stance leg [positive Trendelenburg test]; or if abnormal tiptoe or heel walking was present), sensibility (deemed abnormal if tactile sensibility was reduced), reflexes of the Achilles tendon or patella (deemed abnormal if reduced or not elicited) and the straight-leg-raising test (deemed abnormal if pain provocation in the leg emerged at <; 60°).

The results of the clinical examination were dichotomised as normal or abnormal. The testing procedures and a description of each clinical test were discussed and standardized in meetings with the participating centres before the patients were enrolled.

In order to assess the association between the surgery and the outcome of surgery for the herniated disc, patients who underwent surgery during the observational period reported the date of surgery at follow-up questionnaires. In each questionnaire, the participants were asked whether they had undergone surgery for disc herniation in the period since the last follow-up period, and if so, the patient reported the date of surgery.

### Outcome measure and definition of non-success

The Maine–Seattle Back Questionnaire (MSBQ) was the main outcome measure [27]. The MSBQ is a shortened version of the Roland–Morris Disability Questionnaire that was modified for patients with sciatica and spinal stenosis [28]. The scale is composed of 12 items, each with the answer yes (1) or no (0), achieving a score range of 0–12. The MSBQ assesses disability and functional limits due to sciatic and back pain, and higher scores indicate worse limitations on activity. We have previously reported that the MSBQ is the best measure for distinguishing between success and non-success in sciatica at 1 year of follow-up [29]. Non-success was defined as a MSBQ score ≥ 5.

The secondary outcome measure was the Sciatica Bothersomeness Index (SBI), which is a patient-reported questionnaire that assesses sciatic symptoms [27]. The SBI is a composite of the scores for four symptoms: leg pain (sciatica); numbness or tingling in the leg, foot or groin; weakness in the leg or foot; and back or leg pain while sitting. The scores are in the range of 0–6 for each item, summing to a total score in the range of 0–24, where higher scores indicate worse symptoms. Non-success was defined as a SBI score of ≥ 7 [29].

### Statistical analysis

It has been suggested that for prognostic studies, at least 10 outcome events are required for each factor studied [30]. In the present study, a total of 20 prognostic factors were included. With a sample of 400 patients and poor outcomes for about 50% of the sample, we should have enough power to assess about 20 prognostic factors.

Baseline prognostic variables were analysed using logistic regression with MSBQ ≥ 5 as the primary dependent variable and SBI ≥ 7 as the secondary dependent variable. To compensate for missing items in a questionnaire, the missing item was substituted with the arithmetic mean of the actual respondent’s available item values.

A chi-square test and a Studen’s t-test were used to compare responders and non-responders. The significance level was set at 0.05. The variance inflation factor (VIF) was used to check for multicollinearity and variables with a VIF <; 5 were accepted for inclusion in the model [31].

The results are presented as odds ratios (ORs) with 95% confidence intervals (CIs). Baseline variables from the univariate analysis with a p-value <; 0.10 were included in a multivariate model. The final model controlled for age, sex and baseline variable for the dependent outcome, regardless of the significance in the univariate analysis. The same statistical models were used for 1 and 2 years of follow-ups.

In a backward approach, the non-significant variable with the highest p-value was removed in a stepwise manner until all variables had p <; 0.05. The variable *surgery* (yes/no) was added to the model in the last step, to assess the association between surgery for the herniated disc and the outcome. Variables remaining in the final models were tested for possible interaction effects where the level of interaction was set to p <; 0.01. Nagelkerke R^2^ was used to describe the proportion of variance in the outcome. Data were analysed using the SPSS package (version 18.0) for Windows (SPSS Inc., Chicago, IL).

## Results

### Study sample

In total, 466 patients were included. Four hundred nine patients (88%) responded to the 1-year follow-up questionnaire and 380 (82%) responded to the 2-year follow-up questionnaire. Among the responders at 1 year, 120 (29%) had received surgical treatment. At 2 years, 120 (32%) of the responders were recorded as surgically treated. For patients who were operated, surgery was performed within 3 months of follow-up for 81% of the patients.

### Missing data and non-responders at follow-up

The participants who did not respond to the questionnaire at 1 year were significantly younger, had higher back pain scores and were more frequently positive for the straight-leg-raising test at baseline. The non-responders at 2 years were younger, more often current smokers, had higher back pain scores, more emotional distress and were more frequently positive for the straight-leg-raising test at baseline.

The numbers of missing values replaced at baseline were: SBI, 3 (0.6%); TSK, 26 (5.6%); and MSBQ, 25 (5.4%). The numbers of missing values replaced for the MSBQ were 28 (6.9%) at 1 year and 29 (7.6%) at 2 years. The item in the MSBQ regarding sexual activity had the most missing data: 15 (3.2%) at baseline, 19 (4.6%) at 1 year, and 13 (3.4%) at 2 years.

### Baseline characteristics

The baseline characteristics are presented in Table1. The mean age at baseline was 43.6 years and 58% were males. Most of the patients were employed although only approximately 20% were in work at inclusion. Furthermore, 45% of the sample reported a sciatica episode for the first time. In general, the patients reported high scores on the self-reported questionnaires, in particular for leg pain, disability and emotional distress.

**Table 1:** Baseline characteristics for the total population

	**n = 466**	
**Sociodemographic variables**		
Age years mean (SD)	43.6	(11.5)
Males n (%)	268	(57.5)
Current smoker n (%)	200	(43.3)
Education >; 12 years n (%)	227	(50.1)
Working status		
Working full time n (%)	92	(19.8)
Partly sick leave n (%)	51	(11.0)
Total sick leave, rehabilitation n (%)	234	(50.4)
Disability pension n (%)	33	(7.1)
Other n (%)	54	(11.6)
**Back pain/sciatica history**		
First sciatica episode n (%)	210	(45.3)
Duration back problems <; 1 year n (%)	145	(31.1)
Duration current sciatica episode <; 3 months n (%)	192	(41.4)
**Self-reported health status**		
MSBQ (0–12) mean (SD)	8	(3)
Pain intensity* back (0–100) mean (SD)	42.6	(30.0)
Pain intensity* leg (0–100) mean (SD)	63.2	(28.2)
SBI† (0–24) mean (SD)	14	(5)
Subjective health complaints (0–27) mean (SD)	7.5	(4.5)
Kinesiophobia‡ (13–52) mean (SD)	27	(7)
Emotional distress§ (1–4) mean (SD)	1.58	(0.43)
**Clinical finding**		
Muscular weakness n (%)	203	(44.5)
Reflex reduced or absent n (%)	212	(46.2)
Sensory loss n (%)	273	(59.0)
Straight-leg-raising test <; 60˚ n (%)	267	(58.0)

*Visual analogue scale; †Sciatica Bothersomeness Index; ‡ Tampa Scale for Kinesiophobia; § Hopkins Symptom Check List-25

### Non-success

Patients with non-success (MSBQ ≥ 5) numbered 178 patients (44%) at 1 year and 145 (39%) at 2 years. Among the surgically treated patients, 42 (35%) had non-success at the 1-year follow-up, and 47 (39%) had non-success at the 2-year follow-up. In the non-surgical group, 136 (47%) and 98 (39%) patients had non-success at 1 and 2 years, respectively.

One hundred ninety-four patients (47%) reported non-success, defined as SBI ≥ 7, after 1 year and 159 (42%) after 2 years. Among the surgically treated patients, 36 (30%) reported non-success at the 1-year follow-up and 40 (33%) at the 2-year follow-up. In the non-surgical group, 157 (54%) and 118 (47%) patients reported non-success at 1 and 2 years, respectively.

### Prognostic indicators for non-success defined as MSBQ ≥ 5 (main outcome)

Table2 presents the associations between non-success and all baseline factors both in the univariate analysis and in the final multivariate analyses for the main outcome variable. Higher scores for all self-reported health status variables, shorter education length and longer duration of back problems were significantly associated with non-success at both 1 and 2 years according to the univariate analyses. To assess whether the associations with non-success in the univariate analysis differed when adjustments were made for surgical treatment during follow-up, each baseline variable was adjusted for surgical treatment. This adjustment did not change results. Data are not shown.

**Table 2:** **Factors associated with non-success (MSBQ ≥ 5) at 1 and 2 years. ORs with p <; 0.10 are in*italics*in the univariate analyses**

	**1 year n = 408**					**2 years n = 378**				
	**Univariate**		**Multivariate**			**Univariate**		**Multivariate**		
	**OR**	**95% CI**	**OR**	**95% CI**	**p**	**OR**	**95% CI**	**OR**	**95% CI**	**p**
**Sociodemographic variables**										
Age	1.01	(0.99 *−* 1.03)	1.00	(0.99 *−* 1.02)	0.689	*1.02*	*(1.00 − 1.04)*	1.02	(0.99 *−* 1.04)	0.161
Male sex (ref female)	1.03	(0.70 *−* 1.53)	1.70	(1.06 *−* 2.73)	0.029	0.85	(0.56 *−* 1.30)	1.29	(0.78 *−* 2.15)	0.326
Smoking (ref non-smoking)	*1.99*	*(1.34 − 2.98)*	2.06	(1.31 *−* 3.25)	0.002	*1.51*	*(0.99 − 2.30)*			
Education, years (continuous)	*0.93*	*(0.87 − 0.99)*				*0.92*	*(0.85 − 0.98)*			
Partly sick leave (ref working)	0.84	(0.38 *−* 1.86)				1.50	(0.66 *−* 3.42)			
Complete sick leave, rehabilitation (ref working)	*1.90*	*(1.11 − 3.24)*				*2.18*	*(1.21 − 3.95)*			
Disability pension (ref working)	*2.94*	*(1.23 − 7.01)*				*3.82*	*(1.48 − 9.82)*			
Other (ref working)	*1.99*	*(0.96 − 4.14)*				*2.08*	*(0.94 − 4.60)*			
**Back pain/sciatica history**										
Previous sciatica episodes >; 0 (ref 0)	1.23	(0.83 *−* 1.82)				1.16	(0.76 *−* 1.76)			
Duration back problems ≥ 1 years (ref <; 1 year)	*2.08*	*(1.33 − 3.26)*				*2.48*	*(1.52 − 4.06)*	1.92	(1.11 *−* 3.32)	0.020
Duration current sciatica episode ≥ 3 mos. (ref <; 3 mos.)	1.21	(0.81 *−* 1.80)				*1.69*	*(1.11 − 2.60)*	2.30	(1.40 *−* 3.80)	0.001
**Self-reported health status**										
Maine-Seattle Back Questionnaire (continuous)	*1.26*	*(1.16 − 1.38)*	1.24	(1.11 *−* 1.38)	<;0.001	*1.30*	*(1.19 − 1.44)*	1.28	(1.14 *−* 1.43)	<;0.001
Pain intensity* back (continuous)	*1.02*	*(1.01 − 1.03)*	1.01	(1.01 *−* 1.02)	0.002	*1.02*	*(1.01 − 1.02)*			
Pain intensity* leg (continuous)	*1.01*	*(1.01 − 1.02)*				*1.01*	*(1.01 − 1.02)*			
Sciatica Bothersomeness Index (continuous)	*1.11*	*(1.06 − 1.16)*				*1.11*	*(1.06 − 1.16)*			
Subjective health complaints (continuous)	*1.13*	*(1.07 − 1.82)*	1.09	(1.03 *−* 1.15)	0.003	*1.16*	*(1.10 − 1.23)*	1.10	(1.03 *−* 1.17)	0.003
Kinesiophobia† (continuous)	*1.06*	*(1.03 − 1.09)*				*1.07*	*(1.04 − 1.10)*	1.04	(1.00 *−* 1.08)	0.033
Emotional distress‡ (continuous)	*3.40*	*(2.08 − 5.56)*				*3.31*	*(1.95 − 5.59)*			
**Clinical finding**										
Muscular weakness (ref no weakness)	1.37	(0.92 *−* 2.03)				1.27	(0.84 *−* 1.93)			
Reflex reduced or absent (ref normal reflexes)	*1.41*	*(0.95 − 2.09)*	1.62	(1.03 *−* 2.56)	0.037	1.07	(0.70 *−* 1.62)			
Sensory loss (ref intact sensibility)	1.10	(0.74 *−* 1.64)				1.24	(0.82 *−* 1.90)			
Straight-leg-raising test <; 60° (ref >; 60°)	1.31	(0.88 *−* 1.95)				1.15	(0.76 *−* 1.75)			
**No surgery (ref surgery)**	*1.66*	*(1.07 − 2.58)*	2.97	(1.75 *−* 5.04)	<;0.001	0.98	(0.63 *−* 1.52)	1.32	(0.78 *−* 2.23)	0.308

Multivariate analyses are adjusted for age, sex, and baseline MSBQ. The variable no surgery/surgery was added to the final model. In the multivariable analyses, significant variables, adjusted variables and the variable no surgery/surgery are presented

*Visual analogue scale; †Tampa Scale for Kinesiophobia; ‡ Hopkins Symptom Check List-25

According to the final multivariable regression models, comorbid subjective health complaints was the only prognostic factor significantly associated with non-success at both the 1- and 2-year follow-ups. Other variables independently and significantly associated with non-success at 1 year were male sex, higher intensity of back pain and abnormal reflexes on the clinical examination. Prognostic factors for non-success at 2 years included higher kinesiophobia and longer duration of back pain and sciatica. Adding surgical status to the final models showed non-surgical treatment to be significantly associated with non-success at 1 year (OR = 2.97 [1.75 − 5.04]), but not at 2 years (OR = 1.32 [0.78 − 2.23]). The adjustment variable, the baseline scores of the MSBQ, remained significant in the multivariate models both at 1 and 2 year follow-ups. For the final models, the explained variance assessed by Nagelkerke R^2^ was 26% at both 1 and 2 years. No multicollinearity or interactions were detected for the selected baseline variables and testing for collinearity after the surgery variable was introduced did not change the results. At baseline the correlations assessed by Spearman’s Rho were: Subjective health complaints and kinesiophobia 0.19; subjective health complains and emotional distress 0.59; and kinesiophobia and emotional distress 0.41.

### Prognostic indicators for non-success defined as SBI ≥ 7 (secondary outcome)

Table3 presents the associations between non-success and all baseline factors both in univariate analysis and in the final multivariate analyses for the secondary outcome variable. When adjusting all univariate analyses for surgical treatment during follow-up, only leg pain was significantly associated with non-success at 1 year (p = 0.003). Other data are not shown. In multivariate analyses back pain, comorbid subjective health complaints, muscular weakness at clinical examination, and non-surgical treatment were independently prognostic factors both for the 1 and 2 year results. For the final models, the explained variance assessed by Nagelkerke R^2^ was 26% at 1 year and 20% at 2 years. An interaction effect was detected between surgery and smoking in the 2 year results, p = 0.004.

**Table 3:** **Factors associated with non-success (SBI ≥ 7) at 1 and 2 years. ORs with p <; 0.10 are in*italics*in the univariate analyses**

	**1 year n = 409**					**2 years n = 376**				
	**Univariate**		**Multivariate**			**Univariate**		**Multivariate**		
	**OR**	**95% CI**	**OR**	**95% CI**	**p**	**OR**	**95% CI**	**OR**	**95% CI**	**p**
**Sociodemographic variables**										
Age	*1.03*	*(1.01 − 1.04)*	1.02	(1.00 − 1.04)	0.078	*1.02*	*(1.00 − 1.04)*	1.02	(1.00 *−* 1.04)	0.150
Male sex (ref female)	0.80	(0.54 *−* 1.19)	1.13	(0.71 − 1.82)	0.606	1.06	(0.70 *−* 1.60)	1.36	(0.83 *−* 2.22)	0.218
Smoking (ref nonsmoking)	*1.57*	*(1.06 − 2.34)*				*1.62*	*(1.07 − 2.46)*	1.69	(1.06 *−* 2.68)	0.028
Education, years (continuous)	0.99	(0.92 *−* 1.05)				*0.94*	*(0.88 − 1.01)*			
Partly sick leave (ref working)	1.00	(0.48 *−* 2.09)				0.83	(0.38 *−* 1.82)			
Complete sick leave, rehabilitation (ref working)	1.32	(0.79 *−* 2.21)				1.20	(0.70 *−* 2.07)			
Disability pension (ref working)	*2.24*	*(0.94 − 5.34)*				1.39	(0.56 *−* 3.44)			
Other (ref working)	1.11	(0.54 *−* 2.28)				0.92	(0.43 *−* 2.00)			
**Back pain/sciatica history**										
Previous sciatica episodes >; 0 (ref 0)	*1.47*	*(0.99 − 2.17)*				1.00	(0.67 *−* 1.51)			
Duration back problems ≥ 1 years (ref <;1 year)	*1.97*	*(1.27 − 3.06)*				*1.84*	*(1.16 − 2.93)*			
Duration current sciatica episode ≥ 3 mos. (ref <; 3 mos.)	1.01	(0.68 *−* 1.49)				1.14	(0.75 *−* 1.72)			
**Self-reported health status**										
Maine-Seattle Back Questionnaire (continuous)	*1.10*	*(1.02 − 1.19)*				*1.12*	*(1.03 − 1.22)*			
Pain intensity* back (continuous)	*1.02*	*(1.01 − 1.02)*	1.02	(1.01 *−* 1.03)	<;0.001	*1.02*	*(1.01 − 1.02)*	1.02	(1.01 *−* 1.03)	<;0.001
Pain intensity* leg (continuous)	1.01	(1.00 *−* 1.01)				1.00	(0.99 *−* 1.01)			
Sciatica Bothersomeness Index (continuous)	*1.09*	*(1.05 − 1.14)*	1.06	(1.00 *−* 1.12)	0.037	*1.07*	*(1.03 − 1.12)*	1.03	(0.97 *−* 1.09)	0.346
Subjective health complaints (continuous)	*1.12*	*(1.07 − 1.18)*	1.10	(1.04 *−* 1.16)	0.001	*1.09*	*(1.04 − 1.15)*	1.07	(1.01 *−* 1.13)	0.033
Kinesiophobia† (continuous)	*1.03*	*(1.00 − 1.06)*				1.02	(0.99 *−* 1.06)			
Emotional distress‡ (continuous)	*1.93*	*(1.22 − 3.05)*				*1.88*	*(1.15 − 3.07)*			
**Clinical finding**										
Muscular weakness (ref no weakness)	*1.93*	*(1.30 − 2.88)*	1.70	(1.07 *−* 2.72)	0.026	*1.96*	*(1.29 − 2.97)*	1.94	(1.19 *−* 3.15)	0.008
Reflex reduced or absent (ref normal reflexes)	1.09	(0.74 *−* 1.61)				1.33	(0.88 *−* 2.01)			
Sensory loss (ref intact sensibility)	*1.85*	*(1.24 − 2.76)*				*1.69*	*(1.11 − 2.58)*			
Straight-leg-raising test <; 60° (ref >; 60°)	1.13	(0.76 *−* 1.68)				1.05	(0.69 *−* 1.58)			
**No surgery (ref surgery)**	*2.78*	*(1.76 − 4.37)*	3.81	(2.23 *−* 6.50)	<;0.001	*1.80*	*(1.14 − 2.83)*	2.25	(1.32 *−* 3.81)	0.003

Multivariate analyses are adjusted for age, sex, and baseline SBI. The variable no surgery/surgery was added to the final model. In the multivariable analyses, significant variables, adjusted variables and the variable no surgery/surgery are presented

*Visual analogue scale; †Tampa Scale for Kinesiophobia; ‡ Hopkins Symptom Check List-25

## Discussion

This study shows that 44%–47% of the patients with sciatica who were referred for secondary care had a non-successful outcome at 1 year and 39%–42% at 2 years. For the multivariable models, a high score for comorbid subjective health complaints was the only variable that predicted non-success at both 1 and 2 years. This finding was true for both for the main and the secondary outcome. For the main outcome, males, smokers, patients with higher scores for low back pain and patients who had not undergone surgery had an independent association with non-success at 1 year, but not at 2 years of follow-up. A long duration of back pain and sciatica symptoms and a high level of kinesiophobia were associated with non-success at 2 years. No sciatica-specific clinical findings were associated with non-success, except for a weak association with abnormal tendon reflex at 1 year. For the secondary outcome, muscular weakness at the clinical examination, higher scores for low back pain, and no surgical treatment were associated with non-success after both 1 and 2 years of follow-up. Smoking was also associated with non-success after 2 years of follow-up.

The main strength of this study is the large sample size, the high response rate and the use of imaging to confirm the diagnosis of disc herniation. We used the most precise outcome measures for the current cohort, which in a previous study showed the highest sensitivity and specificity to discriminate between successful outcome or not for sciatica patients [29]. A broad range of potential prognostic variables including several clinical findings, psychological variables and comorbid subjective health complaints were investigated.

A limitation to the internal validity of the study was an incomplete recording of patients who according to the inclusion and exclusion criteria were eligible but for some reason either were not invited or declined to participate. Non-response to the follow-up questionnaires may also have biased the results. Other potential prognostic factors were not investigated in the current study, for example details of imaging findings [20,32-34] and the phenomenon of centralization [35,36] may become important. Lastly, a similar model should be tested in another sciatica sample of sciatica patients with a follow-up period exceeding 1 year.

The Nagelkerke R^2^ values of 20%–26% is consistent with or lower than those of other studies of sciatica cohorts, and indicates that only a small proportion of the variance in the outcome was explained by the included variables [14].

The choice of the main dependent variable, MSBQ ≥ 5, was based on the results of a validation of outcome measurements in the current sciatica cohort [29]. MSBQ with a cut-off of 4.5 had the highest sensitivity and specificity when the global change score was used as an external criterion. The cut-off value for the secondary dependent variable, SBI ≥ 7, was based on the same validation. There is no gold standard for the definition of non-success in patients with sciatica and disc herniation. In 2011, Kamper et al. [37] presented a systematic review of 82 studies of low back pain, including 14 studies of sciatica. They concluded that there is a great variation in how recovery is measured. In two of the studies of sciatica, a composite measure was used that included many different scales of pain and function [20,32]. Eight of the reports did not describe the details of the definition of a good outcome for sciatica. The lack of a gold standard has contributed to the fact that practically no-one has used the same definition or measures of recovery or a successful outcome for sciatica.

The surgery variable was the only variable not recorded at baseline, but only during the follow-up period. This variable is complex because it contains both the decision regarding surgery and the fact that surgery was performed, hence, it was not possible to adjust the variable for symptoms and signs at the time it was decided to operate the patient. This might influence the interpretation of the results of the surgical treatment. However, 81% of the patients who were treated surgically were operated on during the first 3 months of the follow-up period. Consideration might also be given to the fact that the conservative treatments offered could also modify the effects of some of the prognostic factors.

The long-term prognosis in the present study was in accordance with the findings of other studies, but the prognosis for the surgically treated patients in the current study was poorer than that of other studies [9,11]. Additionally, the differences between surgically treated and non-surgically treated patients were smaller than in comparable studies [10,11].

Comorbid subjective health complaints was the only variable associated with non-success at both 1 and 2 years. This variable was associated with both the main and secondary outcome. One possible explanation is that patients with a lower threshold for reporting bodily discomfort report more complaints and have a poorer prognosis. In two Norwegian studies, high scores were associated with reduced function and more complaints in patients with nonspecific low back pain [38] and whiplash [39]. We have previously reported that the patients in the current cohort at baseline reported more comorbid subjective health complaints than the general population and that the number of subjective health complaints nearly doubled in those with persisting sciatica at the 1 year follow-up [40]. In the final model, the significance of emotional distress was not maintained. This is contrary to the results of other studies of sciatica, where emotional distress has been found to be related to pain and disability [17,41]. Emotional distress is also reported to be an important prognostic factor for nonspecific low back pain [42,43]. However, none of those studies tested the influence of comorbidity.

In the present study, in terms of the primary outcome measure, females had better outcomes than males at the 1-year follow-up. This contrasts with the results of the study of Peul [15], in which female sex was a strong predictor for an unsatisfactory outcome at 1 year for patients with sciatica and disc herniation. One possible explanation for the divergent results is that our data were adjusted for subjective health complaints, emotional distress and kinesiophobia. The exclusion criteria in the study of Peul were duration of sciatica symptoms of more than 12 weeks, similar complaints during the previous year, or severe comorbidity. Therefore, our study is probably more representative of the majority of patients with sciatica and disc herniation who are referred for secondary care.

The poor prognosis among smokers is in agreement with the results of some studies on surgically treated patients [19,44], but conflicting results have also been published [14].

Kinesiophobia was an independent prognostic variable for non-success at 2 years. The fear-of-movement/(re)injury model states that the reaction to pain may consist of confrontation in patients with a non-catastrophizing behaviour, and of avoidance in patients with a high catastrophizing behaviour. Pain-related fear may lead to prolonged chronic pain and disability [45] and was prognostic for non-success in patients with nonspecific low back pain after 6 months in two Dutch studies, one of which was a population-based survey [46] and one of which included army workers [47]. In a cross-sectional study of a Swedish population with specific low back pain (defined as disc herniation, isthmic spondylolisthesis or spinal stenosis) attending an orthopaedic clinic, high scores for kinesiophobia were associated with high disability scores [48]. Another cross-sectional Swedish study on patients treated with surgery for disc herniation found that half of the patients suffered from kinesiophobia 10–34 months after the operation and that patients with kinesiophobia were more affected in several other variables as pain, disability and symptoms of depression [49]. Contrary to these findings, fear of movement and pain catastrophizing were not associated with recovery among patients with residual complaints at 3 and 12 months following lumbar disc surgery [50].

Patients who had back pain and sciatica of longer duration at inclusion were about twice as likely to report non-success at 2 years. This is consistent with some studies on surgically treated patients [19,44,51], but inconsistent with studies of conservatively treated patients [20,52]. None of these studies had a follow-up period exceeding 1 year, and therefore they cannot be properly compared with the results of the current study.

The final models for the primary outcome showed that patients who were not treated surgically were nearly three times more likely to report non-success at 1 year, but no significant association was identified between surgical treatment and outcome at 2 years. Most operations were performed during the first 3 months of follow-up. The benefits of surgical treatment decreased with time, which is similar to the results of other studies [3,53,54]. However, when SBI was used as the dependent variable, there was an association between non-success and no surgical treatment after both 1 and 2 years. The SBI is a variable that describes the radicular symptoms of sciatica as pain, sensory symptoms, and paresis. Decision regarding surgical treatment might depend more on the specific sciatic symptoms described in the SBI than on the symptoms related to function in the MSBQ. The interpretation of the interaction effect between smoking and surgery might be that the association between (non-)surgical treatment and (non-)success differs for smokers and non-smokers.

Different prognostic factors were identified for the 1- and the 2-year observations. Comorbidity, kinesiophobia, and duration of symptoms at baseline were associated with non-success at the 2-year follow-up and may indicate that psychosocial factors are more important for the long-term prognosis than sciatica specific symptoms and disability. Fear avoidance and comorbidity are not routinely assessed in consultations for sciatica. Factors and treatments that improve the short-term outcome are important for the patient and for society, but it may be even more important to identify the factors that predict long-term outcomes at an early stage in order to help the patient to solve their problems.

This study identified prognostic factors associated with non-success in sciatica patients. Predictor studies are provided to make estimates of probability and are a supplement for clinicians in their work with the patients [30]. The current results suggest that the prognosis for sciatica patients referred to secondary care is not as good as previously reported and is only slightly better after surgery, and that comorbidity and kinesiophobia should be assessed in patients with sciatica, including surgical candidates.

## Conclusions

The prognostic factors associated with non-success in sciatica patients were comorbidity, smoking, back pain, kinesiophobia, and duration of symptoms. Of the defined sciatica-specific clinical findings, muscular weakness and reduced reflexes were prognostic of non-success. The results indicate that the prognosis for sciatica patients referred to secondary care is not good and is only slightly better after surgery.

This calls for a broader assessment of patients with sciatica than is afforded by the traditional clinical assessment in which mainly the physical symptoms and signs are investigated. The results of the present study may be used to identify subgroups of patients referred to hospital with an increased risk of poor prognosis for sciatica.

## Abbreviations

CI: Confidence interval; CT: Computed tomography; HSCL-25: Hopkins symptom check list-25; MLSS: Maine lumbar spine study; MRI: Magnetic resonance imaging; MSBQ: Maine-seattle back questionnaire; OR: Odds ratio; SBI: Sciatica bothersomeness index; SPSS: Statistical package for the social sciences; TSK: Tampa scale for kinesiophobia; VAS: Visual analogue scale; VIF: Variance inflation factor.

## Competing interests

There are no conflicts of interest to report interest in regard to the present work.

## Authors’ contributions

Conception and design: AJH, LG, MG. Acquisition and data: AJH, LG, AK, DS. Analysis and interpretation of data: AJH, JIB, LG, AK, BN, MG. Drafting the manuscript: AJH. Critical revision of the manuscript: AJH, JIB, LG, AK, DS, BN, MG. Statistical analysis: AJH, LG, MG. All authors read and approved the manuscript.

## Pre-publication history

The pre-publication history for this paper can be accessed here:

http://www.biomedcentral.com/1471-2474/13/183/prepub
